# Effect of Colostrum Components on Early Inflammatory Response, IgG Concentration and Weight Gain in Lambs

**DOI:** 10.3390/ani16060952

**Published:** 2026-03-18

**Authors:** Marina Erm, Maëlle Beck, Joanna Bajzert, Ants Kuks, Tadeusz Stefaniak, Kristel Peetsalu, Toomas Orro

**Affiliations:** 1 Institute of Veterinary Medicine and Animal Science, Estonian University of Life Sciences, 51006 Tartu, Estonia; marina.erm@emu.ee (M.E.); kristel.peetsalu@emu.ee (K.P.); toomas.orro@emu.ee (T.O.); 2 Department of Immunology, Pathophysiology and Veterinary Preventive Medicine, Wrocław University of Environmental and Life Sciences, 50-375 Wrocław, Poland; joanna.bajzert@upwr.edu.pl (J.B.); tadeusz.stefaniak@upwr.edu.pl (T.S.)

**Keywords:** acute phase proteins, colostrum, neonatal lamb, passive transfer, daily weight gain

## Abstract

Newborn sheep rely on passive transfer of immunity via colostrum as well as innate immunity for survival and adaptation to the extrauterine environment. This study examined associations between immunomodulatory constituents of colostrum, namely, immunoglobulin G (IgG), acute-phase proteins (APPs; serum amyloid A (SAA), and haptoglobin) and interleukin-6 (IL-6), and their corresponding concentrations in serum of 2–3-day-old offspring in a cohort of 296 Dorper lambs across three consecutive years. It also assessed the effects of these immunological markers on average daily weight gain (ADWG) during the first four months of life. IgG, APP, and IL-6 concentrations were measured, and mixed linear regression models were used for all analyses. Colostrum and serum concentrations of IgG and IL-6 were positively associated, whereas SAA levels in colostrum were negatively associated with IgG concentrations in serum. Serum IL-6 concentration was negatively associated with colostrum concentrations of IgG and SAA. Serum IgG concentration was positively associated with ADWG, while SAA and IL-6 concentrations were negatively associated with ADWG. These results show the importance of immunomodulating factors in colostrum other than IgG and emphasize the long-term effect of the serum concentrations of these factors on growth performance.

## Simple Summary

Colostrum, the first milk produced after parturition, is an important source of nutrition and antibodies for the newborn, affecting overall development and that of the immune system. Our study investigated associations between levels of important immunological factors in ewe colostrum and the serum of neonatal lambs. Higher antibody levels in colostrum led to higher levels in lambs’ blood. Higher concentrations of serum amyloid A in colostrum were related to lower antibody levels in the lambs’ blood. Higher antibody levels were associated with higher average daily weight gain, while higher levels of inflammatory markers were related to lower daily weight gain over four months. Our findings emphasize that multiple immune factors interact to shape both immediate protection and long-term performance.

## 1. Introduction

The development of the neonatal ruminant’s immune system is highly dependent on colostrum intake, since their synepitheliochorial placenta type prevents the passage of antibodies from the mother to the fetus [1]. The ingestion of colostrum within the first hours of life is essential for absorption of immunoglobulins (Igs), mainly IgG, as antibodies establish the first line of defense against pathogens together with the innate immunity [2]. In addition to providing immunological factors, colostrum contains a variety of fats, proteins, minerals, hormones, growth factors, and enzymes, which fulfill the newborn’s nutritional needs [3]. Thus, sufficient quality and quantity of colostrum are crucial for the development of neonatal ruminants, especially the development of a capable immune system [4]. The quality, notably the IgG concentration, as well as the amount of colostrum available at parturition, can be affected by maternal factors such as diet, age, and environment [5,6]. IgG from colostrum is absorbed by neonatal ruminants through a process called passive transfer of immunity (PTI). The efficiency of PTI is not only influenced by maternal factors, but also by offspring-related factors such as vigor, ability to suckle soon after birth, and the intestinal capacity to absorb immunoglobulins [7,8]. Failure of passive transfer (FPT) is characterized by low serum Ig levels, low body weight, failure to thrive, and, more specifically, early mortality in lambs under one month of age [9]. Lambs with higher birth weights and higher levels of serum Igs are less likely to die during the first month of life [9]. Similarly, IgG levels in lamb serum in the first days of life have shown a significant positive association with average daily weight gain (ADWG) during the first month of life and up to four months of age [10,11].

The relationship between IgG concentrations in colostrum and in offspring serum is well-established in cattle, with calves receiving higher amounts of colostrum IgG showing higher serum IgG concentrations [7,12,13]. However, in lambs, this relationship is less well-understood. Although some studies have confirmed a positive association between IgG levels in the colostrum of ewes and their offspring’s serum [14,15], another study showed no correlation between ewe colostrum and lamb serum IgG concentrations [16]. Moreover, the influence of the ewe’s body condition score, parity, and number of offspring on IgG concentration and PTI remain unclear [14,17,18].

In addition to antibodies, colostrum contains other immunomodulating elements, including acute-phase proteins (APPs) such as haptoglobin (Hp) and serum amyloid A (SAA), and cytokines [19,20]. These proteins are important agents in the systemic inflammatory response and can be used as inflammatory markers in blood serum [21,22]. Interleukin (IL)-6, a pro-inflammatory cytokine, stimulates hepatic production of APPs like Hp and SAA, which help regulate the inflammatory response [23,24]. More specifically, Hp binds free hemoglobin in plasma to prevent oxidative damage and iron loss, while SAA acts as a chemotactic agent for neutrophils and mast cells and facilitates cholesterol transport [24,25]. In calves, colostrum intake leads to increased serum concentrations of IL-6 and SAA, with low levels of APPs found in serum before colostrum ingestion and increased concentrations 24 h after birth [26,27,28]. In lambs, the evidence is more limited, but colostrum SAA concentrations are related to a rise in serum SAA concentrations [15,29]. Notably, elevated serum SAA concentrations in the second week of life were negatively associated with weight gain at 3–4 months of age, indicating that inflammatory processes especially during that time may hinder long-term growth [11,15].

This study aimed to investigate the relationship between the concentrations of immunomodulatory components of colostrum (IgG, APPs, and IL-6; hereafter referred to as “colostrum proteins”) and their corresponding serum levels in 2–3-day-old Dorper lambs. It also explored whether the levels of these components (in colostrum and serum) are associated with future weight gain.

## 2. Materials and Methods

This study consisted of three parts, which were conducted over three consecutive years on the same farm.

### 2.1. Ethics Statement

The study was conducted with permission from the Ethical Evaluation Committee of Animal Experiments of the Estonian Agriculture and Food Board (no. 1.2-17/120 (17 March 2023)).

### 2.2. First Year

A commercial organic sheep farm in Southern Estonia voluntarily participated in this observational study. At the time of the study, approximately 300 breeding ewes, all of the Dorper breed, were kept on the farm. General breeding procedure on this farm occurred once a year in November by joining one genetically suitable ram to a group of 50–60 ewes. Lambing occurred in April and offspring was sold for breeding and food-production purposes in autumn.

Over the winter months, the groups were kept inside two wooden stables with two open sides each and had ad libitum access to water, hay, and wheat silage, as well as minerals (Veskimeister Lammaste mineral 2/1SelPlexB, OÜ Veskimeister, Tõrvandi, Estonia). Other than differing exposure to wind depending on the openings’ direction, both buildings provided the same conditions to the animals, as described below. In the summer months (after lambing had concluded), the sheep were kept on pastures.

In the first year, 67 ewes were included in the study. Of these, 52 gave birth to twins, and 15 to single lambs; thus, a total of 119 lambs were included in the study. Lambing occurred in the group of pregnant ewes in the main stable area with deep bedding to which fresh straw was added 1–2 times per week. Farm workers patrolled the stable at least once every 2 h, and any new newborns and their mothers were then separated from the group into a 1.5 × 1.5 m box with fresh straw bedding and underwent a superficial health check. After 24 h, a second health check was performed to ensure that the offspring had received a sufficient amount of colostrum, showed no signs of illness or abnormalities, and had bonded with the dam. Then, the lambs received chipped ear tags and were released into a second group pen within the same stable, which housed the postpartum ewes with their offspring. As the lambing season progressed, the area for pregnant ewes was reduced in size, and the pen with ewes and lambs was enlarged accordingly.

The last animals were included in the study at the end of April. Two weeks later, the herd was transported to the pasture. The pasture consisted of grassland and shelter in the form of a forest, access to running water, as well as a guard dog. Minerals were available ad libitum, and the herd was checked by the farmers daily. One twin pair included into the study was housed in the 1.5 × 1.5 m box with their dam throughout the first 6 weeks of their lives, since the dam exhibited poor mothering skills and had to be restrained by a person in order to provide milk to the offspring. On one day in early September, the lambs were weighed again on a digital scale.

### 2.3. Second Year

In the following lambing season, overall conditions were the same as in year one, with some exceptions. The animals included were: 20 ewes who gave birth to single lambs, 52 who gave birth to twins, and 2 ewes who gave birth to triplets, yielding a total of 130 lambs for the study. All single lambs were born in the smaller building, while the others were born in the bigger building. After the herd was released onto the pasture, mortality increased, and many lambs developed slowly. Pasteurellosis was diagnosed on the basis of necropsies. The lambs were weighed again on one day in September.

### 2.4. Third Year

In the third year, 36 ewes with twins and 13 with single lambs were included in the study, yielding a total of 77 lambs. Colostrum samples from the ewes were collected using the same procedure as in the two previous years, but from both the left and the right udder halves. IgG concentrations were measured separately in both samples, and the mean of the two was calculated for each ewe.

### 2.5. Sampling

The lambs were weighed on a digital scale (Soehnle Page Profi, Soehnle, Backnang, Germany) in a plastic bucket within 6 h after birth (“birth weight”) and again on a larger digital scale at approximately 4 months (“autumn weight”). Mean (±SD) age at the autumn weighing was 131.3 ± 11.7 days. The lambs’ ADWG was calculated as the difference between autumn weight and birth weight divided by number of days passed between weighings and was used as an outcome variable.

If the birth occurred during the day, one colostrum sample was milked from the ewe into a clean 10 mL tube within 3 h after parturition. In the first 2 years, if the birth occurred during the night, only ewes who lambed after midnight were included, and the sample was taken within 9 h after birth. In year three, only ewes where samples could be obtained within 4 h after parturition were included. Colostrum samples were stored on the farm at −20 °C for up to 7 days before transportation to the laboratory, where they were skimmed by centrifugation at 6149× *g* for 10 min and removal of the fat layer.

Blood samples from the lambs were collected on day 2 of life in the first year (app. 24–36 h postpartum), and on day 3 of life (app. 48–56 h postpartum) in the second and third years. Blood was taken from the jugular vein into sterile Monovettes (Monovette^®^ 9 ml Z, Sarstedt AG & Co KG, Nümbrecht, Germany) with 18 G sterile needles, centrifuged at 5018× *g* for 10 min, after which serum was separated and stored in 1.5 mL aliquots at −20 °C on the farm for up to 7 days until transportation to the laboratory, where they were again stored at −20 °C until analysis.

The total number of samples per year is shown in Table 1.

### 2.6. Laboratory Analysis

The concentrations of IgG in serum and colostrum samples were determined using a sandwich ELISA test. Microplates (Nunc Maxisorp; Thermo Fisher Scientific, Waltham, MA, USA) were coated with polyclonal donkey anti-sheep IgG H&L antibodies (Abcam, Cambridge, UK, ab6895; 5 µg/mL in 0.05 M carbonate buffer pH = 9.6, 100 µL per well, incubation for 2 h at room temperature). After that, plates were washed five times and blocked using Tris buffer saline (TBS; 50 mM Tris; 0.14 M NaCl; pH = 8.0) containing 0.05% Tween 20 (Merck KGaA, Darmstadt, Germany; TBST/200 μL per well). Plates were incubated overnight at 4 °C. Serum samples were diluted 1:200,000 (except 4 samples for which dilution was 1:100,000) with TBST. Colostrum samples were diluted 1:600,000 (except 5 samples for which in two cases dilution was 1: 200,000 and 1:1,200,000 for the others) with TBST. As a standard, sheep IgG calibrator (Abcam, item of 190546) was used. The final concentrations of the calibrator ranged from 500 to 7.8 ng/mL. The calibrator was diluted with TBST. First, 100 µL of the appropriate dilution of samples and calibrator were added to each well, and the plates were incubated at room temperature (22 °C ± 2 °C) for 60 min. The plates were then washed five times with TBST. The dilution of polyclonal donkey anti-sheep IgG H&L antibodies conjugated with HRPO (Abcam, ab6900) was 1:120,000 (100 µL/well). The plates were incubated at room temperature (22 °C ± 2 °C) in the dark for 50 min. The color reaction was developed with supersensitive TMB substrate (Merck; 100 μL/well) in the dark at room temperature for 10 min. The reaction was stopped with 2 M H_2_SO_4_ (P.P.H. Stanlab Sp.J., Lublin, Poland; 100 μL/well). Absorbance was measured at a wavelength of 450 nm by using an ELISA Microplate Reader μQuantum™ (BioTek Instruments, Winooski, VT, USA). The IgG concentration was calculated using BioTek Gen5 Microplate Data Analysis Software (version Gen5 Image+ 2.05.5). All samples and calibrator were assayed in duplicate. The inter-plate control was a randomly selected serum sample that was divided into separate portions at the beginning of the experiment and stored at −80 °C. A new portion of this serum sample was used as a control each time. The control was always diluted 200,000 times in the same dilution series, sequentially 5 serum µL to 995 µL TBST, and then 2 µL to 1998 µL TBST. The control was added in duplicate on each plate, always in the same place (wells H11 and H12). The values read for control always included the midpoints of the standard curve. The intra-assay coefficient of variation (CV) for IgG concentrations in serum samples was 3.9%. The inter-assay CV for IgG concentration in serum samples was 18.3%. The intra-assay CV for IgG concentrations in colostrum whey samples was 3.8%. The inter-assay CV for IgG concentration in colostrum whey samples was 16.3%.

The concentrations of SAA and IL-6 in serum and colostrum were measured using commercial ELISA kits (bovine SAA Phase BE kit, Tridelta Development Ltd., Manhoot, Ireland, as previously used for sheep colostrum and serum in the study by Peetsalu et al. [15]; Sheep IL-6 ELISA kit (Cusabio Biotech, Wuhan, China)) according to the manufacturers’ instructions. For Hp measurements, the colorimetric method developed by Makimura and Suzuki [30] and modified in accordance with the procedure by Alsemgeest et al. [31], by using microtitration plates, and tetramethylbenzine (60.0 mg/L) as the substrate was used. Results were obtained by a spectrophotometer (Magellan Sunrise, Tecan Group Ltd., Männedorf, Switzerland). The minimal detection limits were as follows: 0.3 mg/L for SAA, 60.0 mg/L for Hp and 2.5 ng/L for IL-6. The inter- and intra-assay CVs for SAA and IL-6 in serum and colostrum samples were <15%.

### 2.7. Statistical Analysis

Mixed-effect linear regression models were used to analyze the associations between lambs’ serum protein concentrations and colostrum protein concentrations. The ewe from which the lamb originated was included as a random intercept. Lamb serum concentrations of IgG, SAA, Hp, and IL-6 were included as outcome variables. To achieve normal distribution of the outcome variables, the serum SAA, Hp, and IL-6 concentrations were logarithmically transformed. The following potential confounding variables were included in the models: year of data collection (1, 2, and 3), lamb sex (binary), ewe parity (binary: multiparous, primiparous), number of siblings (single, twin, and triplet), sample hemolysis (binary), and lamb age. Mastitis (binary) was also included as a confounding variable, but it was observed in only three ewes each in years one and two and in no ewes in the third year. Colostrum concentrations of IgG, SAA, Hp, and IL-6 were included as continuous explanatory variables.

Potential interactions and confounding effects were assessed. A stepwise backward elimination procedure was used for model selection, with the significance level set at *p* ≤ 0.05. A variable was retained in the model as a confounder if its removal changed the coefficient of another variable by more than 10%. Linearity between the outcome variables and continuous explanatory variables was assessed, and log-transformation of explanatory variables was performed when necessary to achieve linear relationships between outcome and explanatory variable. Logarithmically transformed colostrum IgG, SAA, and IL-6 continuous explanatory variables were used in the serum IgG model (colostrum log IgG and log SAA). In the serum SAA model, colostrum log IL-6 values were used, and in the serum IL-6 model, colostrum log IL-6, log IgG, and log SAA values were included. Model assumptions were verified using normality and scatter plots of model residuals.

Mixed-effects linear regression was also used to assess associations between lambs’ ADWG over app. 4 months (mean age 131 days) and serum protein concentrations measured at 2–3 days of age and the protein concentrations of their ewes’ colostrum. The same potential confounding variables as in the previous models were included. Lamb serum and the corresponding ewe colostrum protein concentrations were included as continuous explanatory variables. Interaction effects, confounding, and linearity of associations were evaluated. Final models were selected using a stepwise backward elimination procedure, and model assumptions were verified using residual plots.

Pearson’s correlation coefficient r was calculated to investigate whether the concentrations of the measured components were correlated with each other in the colostrum. This analysis was performed by year.

All statistical analyses were performed using Stata^®^ IC14.2 (StataCorp, College Station, TX, USA) and the results were considered significant when *p* ≤ 0.05.

## 3. Results

The laboratory results of serum and colostrum variables are shown in Table 1 and lambs’ weight and ADWG data are summarized in Table 2.

In the first two years, the concentrations of Hp and IgG in the colostrum were positively correlated (r = 0.31 and r = 0.23, respectively; both *p* < 0.05). In the second year, the SAA and Hp concentrations in colostrum were positively correlated (r = 0.42, *p* < 0.001). In the third year, IL-6 and Hp concentrations in colostrum were positively correlated (r = 0.26, *p* = 0.022). SAA and IgG concentrations in colostrum were not correlated in any of the years (see Supplementary Tables S1–S4).

There was a positive association between IgG concentrations in colostrum and offspring serum at 2–3 days of age. Colostrum SAA concentration was negatively associated with serum IgG concentration (Table 3). The number and sex of offspring did not influence this relationship, but the year of sampling played a role.

The colostrum SAA concentration was not associated with the offspring serum SAA concentration at 2–3 days of age, but colostrum IL-6 concentration showed a negative association with serum SAA concentration in the offspring who ingested it (Table 4). Both IgG and SAA concentrations in colostrum were negatively associated with serum IL-6 concentration (Table 4). IL-6 concentrations in colostrum and lamb serum were positively associated (Table 5).

Hp concentrations in colostrum and offspring serum were not associated, but the study years showed a difference from each other, with notably higher Hp concentrations in lamb serum in the first year than in the other two study years.

The serum IgG concentration on day 2–3 of life was positively associated with the ADWG at 131 days of age, while the SAA and IL-6 concentrations were negatively associated with growth (Table 6).

No significant associations were found between concentrations of colostrum proteins and the lamb ADWG.

## 4. Discussion

This study investigated relationships of immunomodulatory components of colostrum with the inflammatory response and future weight gain in lambs. A positive association was found between colostrum and serum IgG concentrations, although with differences across the study years. The colostrum SAA concentration was negatively associated with the serum IgG concentration. While the IL-6 concentrations in colostrum and serum were positively associated, the colostrum IgG and SAA concentrations were negatively associated with the serum IL-6 concentration. Furthermore, high IL-6 concentrations in colostrum were associated with low SAA concentrations in lamb serum. The IgG concentration in serum of 2–3-day-old lambs was positively associated with the 4-month ADWG, whereas the SAA and IL-6 concentrations at this time point were negatively associated with the ADWG.

The mean colostrum IgG concentration of 70.1 ± 41.5 g/L (with substantial differences between the years; see Table 1) in the present study is comparable to previously reported values of 6.2 to 65.4 g/L in different breeds, with meat breeds typically having higher colostrum IgG concentrations than dairy breeds [18]. Scottish lowland meat sheep had colostrum IgG concentrations of 36.78–201.55 g/L [14], and Brazilian Santa Inês meat sheep showed colostrum IgG concentrations of around 37 g/L directly after lambing [17], whereas Akkaraman sheep colostrum contained 60.84 ± 19.41 g/L [32]. Higher colostrum IgG was significantly positively associated with lambs’ serum IgG levels after 2–3 days. The serum IgG concentrations found in this study, 29.7 ± 17.3 g/L, are slightly lower than in Scottish lowland sheep (38.04 ± 16.94 g/L) [14] and Santa Inês sheep (38.04 ± 16.94 g/L) [17], but higher than in Akkaraman crossbred lambs (21.99 ± 11.6 g/L) [33], measured at similar time points.

The synepitheliochorial placenta of ruminants causes calves, lambs, and goat kids to be born hypogammaglobulinemic, relying on adequate colostrum intake to achieve passive immunity [1,34]. Higher and prolonged colostrum supply leads to higher serum IgG concentrations in lambs [35]. In cattle, the process of PTI is well-recognized and monitoring of colostrum quality, i.e., IgG concentration, is a routine part of farm management to prevent FPT, which can lead to increased morbidity and mortality [36]. In lambs, less information is available, and the existing results are not entirely conclusive. Environmental factors, management, and breed may play a role in IgG concentrations in colostrum as well as PTI [16,17,18]. The notable differences in lamb serum and ewe colostrum IgG concentrations across the years in the present study might be due to differing environmental conditions, including nutrition and climate [37]. Similarly, Hp and IL-6 serum concentrations varied across years, indicating that the environment (including climate, nutrition, and early infections) plays an important role in the acquisition of passive immunity. A difference between study years in Hp concentrations in lamb serum has previously been found [15]. Hp concentrations in lambs are high during the first few days of life and then decline over the first month, and Hp levels in the second week of life have been found to be positively associated with ADWG [11,15]. SAA concentrations, however, did not show significant variation across years, as reported previously [11,15]. There is a relationship between SAA and intestinal colonization with microbiota, suggesting that this part of the early inflammatory response in neonates is part of the physiological adaptation process [38,39].

Since the lambs in the present study were allowed to suckle before the colostrum sample was taken, the original IgG concentrations may be higher than those in the sample, due to the reduction in IgG content in colostrum over time [40]. Alves et al. (2015) [17] reported a sharp decline in colostrum IgG concentrations after 6 h postpartum, and in our study, samples were obtained within 6 h (in year 3 within 4 h) after lambing in order to achieve sufficiently accurate results while also prioritizing animal welfare. In the case of twins, more colostrum was consumed by the offspring than in the case of single lambs, presumably leading to a more rapid decline in IgG concentration. In year 3, the exact interval between parturition and sampling was documented and taken into account in the statistical analysis (but proved to have no confounding effect), decreasing this bias. Interestingly, time passed between parturition and sampling was not significantly associated with IgG concentration in colostrum. Another study has previously shown that colostrum composition, specifically the IgG content, is not related to litter size, parity, and interval between parturition and milking [18]. Therefore, the reduction in IgG concentrations in colostrum during the first few hours postpartum is still not clear. No consensus has been reached regarding threshold concentrations of IgG in colostrum and serum to ensure sufficient PTI, since such thresholds depend on many factors, including the breed and the mother–offspring relationship, which affects the suckling ability and amount of ingested colostrum [14,17,18].

These findings highlight that not only IgG, but also environmental and management factors as well as other immunological components of colostrum play a role in passive transfer and early immune defenses of neonatal lambs. In the present study, the SAA concentration in colostrum was negatively associated with IgG concentration in the serum of the offspring. The role of SAA in sheep colostrum has not been sufficiently researched to explain this finding, but we hypothesize that the SAA may affect the absorption of IgG into the bloodstream through the neonatal Fc receptor (FcRn). Inflammatory proteins such as transforming growth factor β1, tumor necrosis factor-α, and IL-1β can upregulate FcRn expression and transcytosis of IgG [41,42]. Thus, SAA, with its anti-inflammatory properties, may be involved in a local process involving inflammatory pathways that may downregulate FcRn expression [43]. Another possibility is that SAA might be able to bind to FcRn and thereby block the transcytosis of IgG, since other APPs, namely fibrinogen and C-reactive protein, have been shown to bind to Fc receptors [44,45].

In newborn foals, plasma transfusions performed to support PTI are associated with an increase in SAA concentration, indicating an important role of SAA in PTI [46]. In human colostrum, SAA1 has been detected (as opposed to SAA3 in ruminant colostrum), but findings by Sack et al. (2018) [47] suggest that the effect of colostrum SAA in the intestine is similar to the effects of cytokines and thereby facilitate local immune protection. In calves, SAA from colostrum does not cross the intestinal barrier into the bloodstream, which, together with the negative association of SAA concentrations in colostrum and serum of the calves, suggests a local protective effect of the colostral SAA [26,27]. In lambs, a positive association between the SAA concentrations in colostrum and serum has been found in previous studies [11,15], suggesting that SAA may cross the intestinal barrier of lambs, although the present study could not confirm this finding.

In the present study, IL-6 concentrations in colostrum and serum were positively associated. This relationship has also been found in calves, further emphasizing the importance of colostrum’s immunological influence beyond Igs [27]. Concentrations of colostrum SAA and IgG were negatively associated with serum IL-6 in the lambs, showing that in lambs, similar to calves, immunomodulatory factors in colostrum affect the immune response of the offspring—and not just by direct transfer, but by interactions between colostrum components and intrinsic immune responses as well. As has been hypothesized for calves, the local protective effect of SAA (and possibly IgG) could reduce the lamb’s own production of pro-inflammatory cytokines in response to inflammatory stimuli [27].

The ADWG at 131 days was associated with neonatal immune marker concentrations—IgG positively, as has been shown in previous studies in lambs and calves [10,11,33,48], and SAA and IL-6 negatively, as has been observed in calves [49,50]. Since growth depends on many factors with greater influence than neonatal subclinical inflammatory responses, the effect sizes were expectedly small: 1 mg/L difference in SAA concentration at 2–3 days of age led to a weight difference of 5.24 g at 131 days of age. However, the individual animals showed considerable variations in SAA concentrations (SD 70 mg/L), where a difference in this SD in SAA concentrations corresponded to a weight difference of 360 g at 131 days of age. The differences between the low-SAA lambs and the high-SAA lambs were more substantial. The effect size of this relationship is considerably bigger at 6–12 days of life [15].

In addition to the abovementioned caveats, another limitation of this study was the overrepresentation of the second year in the dataset. Controlling for the effect of the year in the model allowed us to account for potential inter-annual variations. These also included slight differences in the timing of colostrum sampling (as mentioned above), climatic conditions, and timing of the blood sampling. Since no interaction was found between years and colostrum and serum associations in the statistical models, the yearly differences in protein concentrations were unlikely to have affected the associations found in this study. Absorption of IgG and, therefore, successful PTI is not only dependent on IgG concentration in colostrum and the timing of colostrum feeding, but also the volume ingested [51]. In the present study, ingested volume was not measured, as the lambs were not handled before suckling for animal welfare reasons. Since the statistical models showed a significant association between colostrum and lamb serum IgG concentrations, the influence of colostrum volume on serum IgG seems to be at least less significant than the colostrum quality, if not negligible.

Additionally, the in-house IgG-ELISA exhibited inter-assay CVs of 18.3% for serum and 16.3% for colostrum. Although these values are typical for in-house assays involving complex biological matrices, they are higher than the ideal threshold of 15%. This variability was likely due to minor fluctuations in ambient temperature and timing over the five-month testing period, limiting the precision of absolute IgG quantification and possibly impairing the accuracy of regression coefficients, potentially leading to underestimated effect sizes and wider standard errors. Nevertheless, given the consistent use of internal controls and replicates across all plates, we believe that the observed biological trends remain meaningful and reliable because they depend on consistent sample ranking rather than exact absolute values. Possible sources of bias from the IgG analysis are controlled for in the models—year and ewe—which further means that the influence of the high inter-assay CV rather leads to an under- than an overestimation of the effects.

Furthermore, mastitis may affect the concentrations of IgG and inflammatory markers (such as APPs) in colostrum [52]. However, since only three ewes exhibited clinical signs of mastitis per study year, this finding showed no statistical effect. Microbiological tests for subclinical mastitis were not performed, and mastitis was diagnosed on the basis of clinical signs alone, so the effect of subclinical mastitis could not be evaluated in the present study.

## 5. Conclusions

The results of this study show that colostrum IgG concentrations are positively associated with serum IgG levels in lambs regardless of parity, sex, or litter size, but the immune regulation provided by colostrum extends beyond IgG alone. Colostrum IgG, SAA, and IL-6 showed complex relationships with neonatal serum markers, including a negative association of colostrum SAA levels with serum IgG concentration. Early-life serum IgG concentrations were positively associated with later weight gain, while early-life serum SAA and IL-6 concentrations showed negative associations, suggesting that early inflammatory activity has long-term effects. Overall, these results emphasize the importance of other immunomodulatory factors in addition to IgG for PTI and lambs’ health and performance.

## Figures and Tables

**Table 1:** Concentrations of immunoglobulin G (IgG), serum amyloid A (SAA), haptoglobin (Hp) and interleukin-6 (IL-6) in lamb serum and sheep colostrum by study year.

	IgG (g/L) Mean ± SD	SAA (mg/L) Median (Interquartile Range)	Hp (mg/L) Median (Interquartile Range)	IL-6 (ng/L) Median (Interquartile Range)
**Serum**				
Year 1 (n = 99)	26.2 ± 18.5 ^a^	43.2 ^a^ (18.9–77.2)	281.2 ^a^ (217.2–400.5)	7.6 ^a^ (3.8–30.1)
Year 2 (n = 120)	36.1 ± 18.1 ^ab^	60.7 ^a^ (23.6–114.7)	175.2 ^b^ (154.4–210.0)	13.2 ^ab^ (5.5–33.6)
Year 3 (n = 77)	24.1 ± 9.6 ^ac^	34.9 ^a^ (18.8–98.1)	186.5 ^b^ (163.7–221.0)	6.1 ^ac^ (3.4–17.9)
All years (n = 296)	29.7 ± 17.3	43.7 (20.4–94.6)	201.7 (166.0–256.0)	9.8 (4.1–26.8)
**Colostrum**				
Year 1 (n = 58)	75.2 ± 46.1 ^a^	5.5 ^a^ (2.1–15.0)	399.7 ^a^ (332.1–452.7)	10.8 ^a^ (4.4–40.0)
Year 2 (n = 71)	55.4 ± 35.3 ^b^	5.6 ^a^ (2.2–16.2)	187.1 ^b^ (146.7–227.0)	16.5 ^a^ (5.4–37.0)
Year 3 (n = 47)	85.9 ± 37.2 ^a^	2.2 ^a^ (1.1–9.4)	294.9 ^c^ (247.0–321.0)	40.8 ^a^ (6.1–103.8)
All years (n = 176)	70.1 ± 41.5	4.2 (1.8–13.9)	269.2 (196.8–380.0)	14.5 (5.2–47.3)

Different superscript letters indicate statistically significant differences (Bonferroni-adjusted *p* < 0.05) between years within columns (serum and colostrum analyzed separately).

**Table 2:** Lamb weights and average daily weight gain (ADWG) overall and by study year.

	Birth Weight (kg) Mean ± SD	Autumn Weight (kg) Mean ± SD	Age at Autumn Weighing (Day) Mean ± SD	ADWG (g/Day) Mean ± SD
Year 1 (n = 91)	3.4 ± 0.7	23.1 ± 4.1	135.1 ± 2.5	146.1 ± 27.7 ^a^
Year 2 (n = 112)	3.5 ± 0.8	22.1 ± 5.0	118.5 ± 3.9	156.6 ± 36.6 ^a^
Year 3 (n = 73)	3.6 ± 0.9	32.8 ± 6.0	145.9 ± 4.2	200.1 ± 38.6 ^b^
All years (n = 276)	3.5 ± 0.8	25.3 ± 6.8	131.3 ± 11.7	164.6 ± 40.7

Different superscript letters indicate statistically significant differences (Bonferroni-adjusted *p* < 0.05) between years within each column.

**Table 3:** Associations of serum immunoglobulin G (IgG, g/L) concentration in 2–3-day-old lambs (*n* = 258) with colostrum protein concentrations. Results of the mixed regression model with ewe as the random intercept.

Variable	*n*	Coef.	95% CI of Coef.	*p*-Value	Wald Test *p*-Value
Age (days)		−3.26	−15.36; 8.84	0.530	
**Year:**					<0.001
1	61	0			
2	120	13.79	0.18; 27.40	0.047	
3	77	−2.09	−15.38; 11.20	0.758	
Colostrum IgG, log (g/L)		3.40	0.53; 6.26	0.020	
Colostrum SAA, log (mg/L)		−1.84	−3.11; −0.57	0.004	
**Intercept**		20.25	3.06; 37.45	0.021	

CI—confidence interval; SAA—serum-amyloid A. Likelihood ratio *p* < 0.001 for random effect.

**Table 4:** Associations of serum amyloid A (SAA, logarithmically transformed, mg/L) concentration in the serum of 2–3-day-old lambs (*n* = 258) with colostrum protein concentrations. Results of the mixed regression model with ewe as the random intercept.

Variable	*n*	Coeff.	95% CI of Coeff.	*p*-Value	Wald Test *p*-Value
Age (days)		−0.10	−0.98; 0.78	0.824	
**Year:**					0.217
1	61	0			
2	120	0.64	−0.34; 1.61	0.201	
3	77	0.38	−0.57; 1.33	0.434	
Colostrum IL-6, log (ng/L)		−0.13	−0.24; −0.03	0.014	
**Intercept**		3.94	2.94; 4.93	<0.001	

CI—confidence interval; IL-6—interleukin-6. Likelihood ratio *p* < 0.001 for random effect.

**Table 5:** Associations of interleukin-6 (IL-6, logarithmically transformed, ng/L) in the serum of 2–3-day-old lambs (*n* = 258) with colostrum protein concentrations. Results of the mixed regression model with ewe as the random intercept.

Variable	*n*	Coeff.	95% CI of Coeff.	*p*-Value	Wald Test *p*-Value
Age (days)		0.08	−0.56; 0.72	0.804	
**Year:**					<0.001
1	61	0			
2	120	0.23	−0.48; 0.94	0.530	
3	77	−0.70	−1.4; 0.003	0.051	
Colostrum IL-6, log (ng/L)		0.52	0.43; 0.60	<0.001	
Colostrum IgG, log (g/L)		−0.36	−0.54; −0.19	<0.001	
Colostrum SAA, log (mg/L)		−0.13	−0.19; −0.06	<0.001	
**Model intercept**		2.53	1.60; 3.46	<0.001	

CI—confidence interval; SAA—serum amyloid A; IgG—immunoglobulin G. Likelihood ratio *p* < 0.001 for random effect.

**Table 6:** Associations of average daily weight gain (ADWG) (g/day) of 131-day-old lambs (*n* = 277) with serum inflammatory marker concentrations (at 2–3 days of age). Results of the mixed regression model with ewe as the random intercept.

Variable	*n*	Coeff.	95% CI of Coeff.	*p*-Value	Wald Test *p*-Value
**Year:**					<0.001
1	91	0			
2	112	3.90	−6.22; 14.02	0.451	
3	73	46.57	36.21; 56.94	<0.001	
IgG (g/L)		0.28	0.05; 0.52	0.017	
SAA (mg/L)		−0.04	−0.07; −0.006	0.019	
IL-6 (ng/L)		−0.18	−0.35; −0.01	0.035	
**Number of siblings:**					<0.001
Single	41	0			
Twin	229	−35.53	−46.27; −24.79	<0.001	
Triplet	6	−44.87	−76.71; −13.02	0.006	
**Ewe parity:**					0.036
Multiparous	210	0			
Primiparous	66	−14.16	−23.7; −4.62	0.004	
**Intercept**		182.45	167.88; 197.02	<0.001	

CI—confidence interval; SAA—serum amyloid A; IgG—immunoglobulin G, IL-6—interleukin-6. Likelihood ratio *p* < 0.001 for random effect.

## Data Availability

The original contributions presented in this study are included in the article/Supplementary Material. Further inquiries can be directed to the corresponding author.

## Supplementary Materials

The following supporting information can be downloaded at https://www.mdpi.com/article/10.3390/ani16060952/s1, Table S1: Pearson correlation coefficients of colostrum components in all three study years combined (*n* = 258); Table S2: Pearson correlation coefficients of colostrum components, first year only (*n* = 61); Table S3: Pearson correlation coefficients of colostrum components, second year only (*n* = 120); Table S4: Pearson correlation coefficients of colostrum components, third year only (*n* = 77).

## Abbreviations

The following abbreviations are used in this manuscript:

ADWG	Average daily weight gain
APP	Acute phase protein(s)
FcRn	Neonatal Fc receptor
FPT	Failure of passive transfer of immunity
Hp	Haptoglobin
IgG	Immunoglobulin G
IL-6	Interleukin-6
PTI	Passive transfer of immunity
SAA	Serum amyloid A
