# Supplementary material for: Effect of Colostrum Components on Early Inflammatory Response, IgG Concentration and Weight Gain in Lambs
Source: Animals (Basel). 2026 Mar 18;16(6):952. doi: 10.3390/ani16060952 (PMC13023249; doi:10.3390/ani16060952)
Supplement: Supplementary file 1 [file animals-16-00952-s001.zip › animals-4139565-supplementary.pdf]

Article

Effect of Colostrum Components on Early Inflammatory Response, IgG Concentration and Weight Gain in Lambs

Marina Erm [1], Maëlle Beck [1,*], Joanna Bajzert [2], Ants Kuks [1], Tadeusz Stefaniak [2], Kristel Peetsalu [1] and Toomas Orro [1]

[1] Institute of Veterinary Medicine and Animal Science, Estonian University of Life Sciences, 51006 Tartu, Estonia; marina.erm@emu.ee (M.E.); ants.kuks@gmail.com (A.K.); kristel.peetsalu@emu.ee (K.P.); toomas.orro@emu.ee (T.O.)

[2] Department of Immunology, Pathophysiology and Veterinary Preventive Medicine, Wrocław University of Environmental and Life Sciences, 50-375 Wrocław, Poland; joanna.bajzert@upwr.edu.pl (J.B.); tadeusz.stefaniak@upwr.edu.pl (T.S.)

* Correspondence: maelle.beck@emu.ee

Abbreviations and units:

IgG – immunoglobulin G, g/L

IL-6 – interleukin-6, ng/L

Hp – haptoglobin, mg/L

SAA – serum amyloid A, mg/L

Table S1. Pearson correlation coefficients of colostrum components in all three study years combined (n = 258).

	IgG	SAA	Hp	IL-6
IgG	1			
SAA	-0.10<br>(p = 0.079)	1		
Hp	**0.35**<br>(p < 0.001)	0.07<br>(p = 0.239)	1	
IL-6	**0.24**<br>(p < 0.001)	-0.07<br>(p = 0.263)	**0.15**<br>(p = 0.016)	1

Table S2. Pearson correlation coefficients of colostrum components, first year only (n = 61).

	IgG	SAA	Hp	IL-6
IgG	1			

SAA	0.05<br>(p = 0.647)	1		
Hp	**0.31**<br>(p = 0.002)	0.03<br>(p = 0.762)	1	
IL-6	0.25<br>(p = 0.057)	-0.01<br>(p = 0.939)	0.05<br>(p = 0.726)	1

Table S3. Pearson correlation coefficients of colostrum components, second year only (n = 120).

	IgG	SAA	Hp	IL-6
IgG	1			
SAA	-0.08<br>(p = 0.383)	1		
Hp	**0.23**<br>(p = 0.010)	0.42<br>(p < 0.001)	1	
IL-6	0.11<br>(p = 0.243)	-0.06<br>(p = 0.545)	0.01<br>(p = 0.928)	1

Table S4. Pearson correlation coefficients of colostrum components, third year only (n = 77).

	IgG	SAA	Hp	IL-6
IgG	1			
SAA	-0.09<br>(p = 0.431)	1		
Hp	0.13<br>(p = 0.281)	-0.045<br>(p = 0.699)	1	
IL-6	0.20<br>(p = 0.08)	-0.10<br>(p = 0.380)	**0.26**<br>(p = 0.0322)	1
